# Mental health disparities in young adults with arrest history: a survey-based, cross-sectional analysis

**DOI:** 10.1186/s40352-023-00257-2

**Published:** 2024-01-02

**Authors:** Onur Baser, Katarzyna Rodchenko, Yixuan Zeng, Amy Endrizal

**Affiliations:** 1 Department of Economics, Bogazici University, Istanbul, Türkiye; 2 Graduate School of Public Health, City University of New York, New York, NY USA; 3 University of Michigan Medical School, Ann Arbor, MI USA; 4 Columbia Data Analytics, New York, NY USA; 5 Department of Biostatistics, University of Michigan, Ann Arbor, MI USA

**Keywords:** Mental health, Arrest history, Criminal justice, Young adults, Disparities

## Abstract

**Background:**

Over 4.53 million arrests were made in 2021 in the United States. People under 26 years of age were more likely to be arrested than older people. Although mental health disparities are prominent in the incarcerated population, the subject has not been closely examined among young adults specifically.

**Objectives:**

This study examines how criminal justice involvement, specifically arrests, affects the mental health of adults between 18 and 25 years of age.

**Methods:**

We analyzed secondary data using the 2021 National Survey on Drug Use and Health (NSDUH). The study used a subsample of 13,494 people aged 18 to 25 years, including 7,330 women and 6,164 men. History of arrest was the key independent variable. Depression, serious mental illness (SMI), substance use, suicidal ideation, and suicide attempt were the outcome variables. We performed five multivariate logistic regression models for each outcome variable, controlling for race/ethnicity, income, and education level for men and women separately.

**Results:**

Of 13,494 respondents, 6.63% had a history of arrest. Among young women, a history of arrest was associated with significantly higher adjusted odds ratios for all mental health concerns. Most notably, a history of arrest increased the likelihood of substance use by a factor of 15.19, suicide attempts by 2.27, SMI by 1.79, suicidal ideation by 1.75, and depression by 1.52. Among young men, a history of arrest was associated with increased adjusted odds ratios (AORs) for substance use (AOR, 13.37; *p* < .001), suicidal ideation (AOR, 1.45; *p* = .011), and suicide attempt (AOR, 1.82; *p* = .044).

**Conclusions:**

We found a strong relationship between young people having an arrest history and mental health concerns. More specifically, a history of arrest was associated with all mental health concerns among young women, while it was associated with only substance use and suicide among young men. Providing arrestees with appropriate mental health care would benefit them and the criminal justice system by decreasing the odds of recidivism.

## Background

The United States has the highest incarceration rate globally, with 644 incarcerated individuals per 100,000 population as of 2021, costing the country approximately $182 billion annually (Prison Policy Initiative, 2023; Wagner & Rabuy, 2017). Moreover, approximately 5.4 million adults were under the supervision of US correctional systems at the end of 2021 (Carson & Kluckow, 2023). Criminal justice involvement is negatively associated with a variety of health outcomes (Barnert et al., 2016). According to the Treatment Advocacy Center, compared with the general population, people with mental illness are more likely to get arrested, are incarcerated for longer periods, and cost more to house (Treatment Advocacy Center, 2016).

Additionally, researchers found that inmates experience high rates of mental health issues while imprisoned and long after their release from the institution (Bebbington et al., 2021). According to the US Department of Justice, among all state and federal prisoners, 41% (66% of whom were women) had a history of mental illness in 2016. Moreover, in the 2016 Survey of Prison Inmates, 27% of state prisoners reported being told they had a major depressive disorder, 22% reported an anxiety disorder, and 14% reported post-traumatic stress disorder (Maruschak et al., 2021). According to The National Alliance on Mental Illness, 63% of people with mental illness do not receive treatment while incarcerated. Moreover, about 4,000 people with serious mental illness are held in solitary confinement inside US prisons, where suicide is the leading cause of death (The National Alliance on Mental Illness, 2023).

Health studies have tended to concentrate on incarceration and adult outcomes rather than on arrest alone, since the former may indicate more extensive contact with the criminal justice system. However, the arrest rate is almost five times higher than the incarceration rate: 3,011 arrests per 100,000 population (Statista, 2023a) versus 630 incarcerated in prison or local jail per 100,000 population (Minton et al., 2021) in 2019. Therefore, the correlation between involvement in the criminal justice system and health may be underestimated in research that addresses only incarceration rates rather than arrest records. Over 4.53 million arrests were made in 2021 in the United States (Statista, 2023b). Moreover, people under 26 years of age were more likely to be arrested than older people (RAND Corporation, 2019). Although mental health disparities have been found in the incarcerated population, the subject has not been closely examined among young adults. Therefore, this study aims to narrow this lack in literature by exploring relationships between a history of arrest and mental health concerns among men and women ages 18 to 25 in the United States.

## Methods

The 2021 National Survey on Drug Use and Health was used for this study. The survey, conducted annually by the Substance Abuse and Mental Health Services Administration (SAMHSA) among the noninstitutionalized US population aged 12 or older, provides nationally representative data including tobacco use, substance use, and mental health issues. Research reveals that self-rated health is a reliable assessment of current and future health, gender, and age (Benjamins et al., 2004; Benyamini et al., 2003; Idler et al., 2004). Data were collected via in-person interviews and online surveys using the same questionnaire (Center for Behavioral Health Statistics and Quality, 2022).

A subsample of 13,494 people (7,330 women and 6,164 men) between 18 and 25 years of age was used for this study. Among them, 7.20% (6.63% of women and 7.88% of men) had a history of arrest. Of this group, 53.36% of women and 54.34% of men identified as non-Hispanic white; 12.19% of women and 11.34% of men identified as black or African American; 21.62% of women and 20.36% of men as Hispanic or Latinx; and 12.83% of women and 14.07% of men identify as other race/ethnicity or as multiracial. Among women, 10.49% reported their educational achievement as less than a high school diploma; among men, this was 13.64%. Moreover, 29.02% of women and 22.63% of men had a household income below 100% of the federal poverty income limit (Table 1).

**Table 1:** Descriptive statistics of study subsample of women and men, 18–25 years (N = 13,494)

	Women (%)	Men (%)
Proportion	54.32	45.58
History of arrest	6.63	7.88
Median age	21–23	21–23
Race/ethnicity		
White	53.36	54.34
Black/African American	12.19	11.34
Hispanic/Latinx	21.62	20.36
Multiracial/other	12.83	14.07
Education achieved		
High school diploma	89.51	86.36
No high school diploma	10.49	13.64
Income level		
Above poverty threshold	70.98	77.37
Below poverty threshold	29.02	22.63
Mental health concern		
Depression	22.48	12.31
SMI	13.68	6.99
Substance use	2.63	3.44
Suicidal ideation	14.00	9.51
Suicide attempt	2.66	1.59

### Measures

#### Independent variable

The independent variable is a history of arrest, based on the survey question, “Have you ever been arrested and booked for breaking the law?” Responses were coded as 0 = no history of arrest and 1 = a record of arrest.

#### Control variables

Three control variables were used. These included, first, a race/ethnicity variable, where the non-Hispanic white racial group was used as the reference group; African American, Hispanic/Latinx, and multiracial or other racial groups were created as dichotomous variables. Second, the education-level variable was coded as 0 = high school diploma or higher and 1 = less than a high school diploma. Finally, the income-level variable was coded as 0 = above or equal to 100% of the federal poverty threshold and 1 = below 100% of the threshold.

#### Outcome variables

Five outcome variables—depression, serious mental illness (SMI), substance use, suicidal ideation, and suicide attempt—were used in the analysis. First, depression was assessed using DSM-IV criteria for major depressive episodes (Center for Behavioral Health Statistics and Quality, 2022). The variable was coded as 1 if the criteria for major depressive episode were met in the past year and 0 if otherwise. Second, the SMI variable was assessed according to Sect. 1912(c) of the Public Health Service Act, as amended by Public Law 102–321, defining “adults with serious mental illness” (Center for Behavioral Health Statistics and Quality, 2022). The variable was coded 1 if the SMI definition criteria were met and 0 if otherwise. Third, the substance use variable was assessed based on the question, “Have you ever received alcohol or drug treatment?” The variable was coded as 1 if the answer was “Yes” and 0 otherwise. Fourth, the suicidal ideation variable was assessed by the question, “Have you seriously thought about killing yourself in the past year?” and was coded 1 if the respondent had seriously thought about suicide and 0 if there were no serious suicidal thoughts. Fifth, the suicide attempt variable was based on the question, “Have you attempted to kill yourself in the past year?” The variable was coded 1 if there was a suicide attempt 0 for no suicidal attempt.

### Statistical analysis

R was used to conduct statistical analysis. Multivariate logistic regression models for each mental health outcome were performed separately for women and men between 18 and 25 years old. Controlling for demographic factors, 10 multivariate logistic regression analyses (five for each population group) were conducted to determine whether a history of arrest was significantly related to a range of mental health concerns. The independent and outcome variables were coded dichotomously.

## Results

The study investigated mental health disparities among young women and men with a history of arrest. Descriptive statistics are shown in Tables 2 and 3. Among women with an arrest history, 51.64% identified as non-Hispanic white, 16.12% as Black or African American, 19.7% as Hispanic or Latina, and 12.54% as multiracial or other. Among men, 53.7% identified as non-Hispanic white, 15.84% as Black or African American, 18.11% as Hispanic or Latino, and 12.35% as multiracial or other. Both women and men with an arrest history more often had education achievement of less than a high school diploma and lived below the poverty threshold compared with their counterparts without an arrest history.

**Table 2:** Descriptive statistics of young women (18–25 years) with and without an arrest history (2021)

Variable	No Arrest History (%)	With Arrest History (%)	*P* Value
Race/ethnicity			0.137
White	53.48	51.64	
Black/African American	11.91	16.12	
Hispanic/Latinx	21.76	19.70	
Multiracial/other	12.85	12.54	
Education achieved			
High school diploma	89.95	83.28	< 0.001*
No high school diploma	10.05	16.72	
Income level			< 0.001*
Above poverty threshold	71.57	62.69	
Below poverty threshold	28.43	37.31	
Mental health concern			
Depression	22.16	29.25	0.003*
SMI	13.34	20.90	< 0.001*
Substance use	1.73	21.49	< 0.001*
Suicidal ideation	13.64	21.49	< 0.001*
Suicide attempt	2.50	5.97	< 0.001*

*p < 0.05 is considered as significant

**Table 3:** Descriptive statistics of young men with and without an arrest history (2021)

Variable	No Arrest History (%)	With Arrest History (%)	*P* Value
Race/ethnicity			0.008*
White	54.39	53.7	
Black/African American	10.95	15.84	
Hispanic/Latino	20.55	18.11	
Multiracial/other	14.22	12.35	
Education achieved			
High school diploma	87.43	73.87	< 0.001*
No high school diploma	12.57	26.13	
Income level			0.396
Above poverty threshold	77.51	75.72	
Below poverty threshold	22.49	24.28	
Mental health concern			
Depression	12.21	13.58	0.416
SMI	6.83	8.85	0.114
Substance use	1.95	20.78	< 0.001*
Suicidal ideation	9.25	12.55	0.021*
Suicide attempt	1.48	2.88	0.029*

*p < .05

The results of five binary logistic regression models are shown for women (Table 4) and men (Table 5). Among young women, an arrest history was associated with significantly higher adjusted odds ratios for all mental health concerns. Most notably, an arrest history increased the likelihood of substance use by a factor of 15.19 and suicide attempts by a factor of 2.27. Moreover, the women who encountered the criminal justice system were 1.79 times more likely to have SMI, 1.75 times more likely to think about suicide, and 1.52 times more likely to have depression. Among young men, a history of arrest was associated with increased adjusted odds ratios (AORs) for substance use (AOR, 13.37; *p* < .001), suicide ideation (AOR, 1.45; *p* = .011), and suicide attempt (AOR; 1.82; *p* = .044).

**Table 4:** Binary logistic regression model results of mental health concerns for young women (2021)

Model	AOR [95% Confidence Interval]	*P* Value
Model 1: Depression		
Arrest history	1.52 [1.19, 1.94]	< 0.001*
Black/African American	0.54 [0.44, 0.65]	< 0.001*
Hispanic/Latina	0.74 [0.64, 0.85]	< 0.001*
Multiracial/other	0.90 [0.76, 1.06]	0.208
Below poverty threshold	0.82 [0.73, 0.93]	0.003*
No high school diploma	0.87 [0.72, 1.05]	0.147
Model 2: SMI		
Arrest history	1.79 [1.35, 2.35]	< 0.001*
Black/African American	0.49 [0.38, 0.64]	< 0.001*
Hispanic/Latina	0.70 [0.58, 0.84]	< 0.001*
Multiracial/other	1.01 [0.83, 1.23]	0.925
Below poverty threshold	0.89 [0.76, 1.03]	0.132
No high school diploma	0.88 [0.69, 1.10]	0.264
Model 3: Substance use		
Arrest history	15.19 [10.95, 20.96]	< 0.001*
Black/African American	0.28 [0.14, 0.50]	< 0.001*
Hispanic/Latina	0.54 [0.35, 0.80]	0.003*
Multiracial/other	0.71 [0.43, 1.11]	0.145
Below poverty threshold	1.51 [1.10, 2.06]	0.010*
No high school diploma	2.18 [1.48, 3.15]	< 0.001*
Model 4: Suicidal ideation		
Arrest history	1.75 [1.33, 2.29]	< 0.001*
Black/African American	0.72 [0.57, 0.91]	0.006*
Hispanic/Latina	0.89 [0.75, 1.05]	0.172
Multiracial/other	1.13 [0.93, 1.38]	0.205
Below poverty threshold	0.90 [0.77, 1.04]	0.16
No high school diploma	1.15 [0.93, 1.41]	0.198
Model 5: Suicide attempt		
Arrest history	2.27 [1.36, 3.58]	< 0.001*
Black/African American	1.54[0.99, 2.32]	0.048*
Hispanic/Latina	1.52 [1.07, 2.16]	0.019*
Multiracial/other	1.32 [0.84, 2.03]	0.216
Below poverty threshold	1.15 [0.84, 1.55]	0.383
No high school diploma	2.38 [1.66, 3.55]	< 0.001*

*p < .05

*Abbreviations*: AOR, adjusted odds ratio; CI, confidence interval

**Table 5:** Binary logistic regression model results of mental health concerns for young men (2021)

Model	AOR [95% CI]	*P* Value
Model 1: Depression		
Arrest history	1.19 [0.90, 1.55]	0.217
Black/African American	0.64 [0.48, 0.83]	0.001*
Hispanic/Latino	0.86 [0.70, 1.04]	0.129
Multiracial/other	0.78 [0.61, 0.99]	0.042*
Below poverty threshold	0.99 [0.82, 1.19]	0.885
No high school diploma	0.75 [0.58, 0.95]	0.019*
Model 2: SMI		
Arrest history	1.39 [0.99, 1.92]	0.051
Black/African American	0.49 [0.33, 0.72]	< 0.001*
Hispanic/Latino	0.77 [0.59, 0.99]	0.046*
Multiracial/other	0.77 [0.56, 1.03]	0.085
Below poverty threshold	1.00 [0.78, 1.26]	0.979
No high school diploma	0.78 [0.56, 1.06]	0.123
Model 3: Substance use		
Arrest history	13.37 [9.95, 17.97]	< 0.001*
Black/African American	0.63 [0.37, 1.02]	0.075
Hispanic/Latino	0.83 [0.55, 1.21]	0.345
Multiracial/other	1.37 [0.91, 2.00]	0.12
Below poverty threshold	0.81 [0.56, 1.16]	0.262
No high school diploma	1.14 [0.78, 1.64]	0.481
Model 4: Suicidal ideation		
Arrest history	1.45 [1.08, 1.91]	0.011*
Black/African American	0.65 [0.47, 0.87]	0.006*
Hispanic/Latino	0.76 [0.60, 0.95]	0.017*
Multiracial/other	0.81 [0.62, 1.05]	0.113
Below poverty threshold	1.12 [0.91, 1.36]	0.29
No high school diploma	0.87 [0.67, 1.12]	0.304
Model 5: Suicide attempt		
Arrest history	1.82 [0.98, 3.15]	0.044*
Black/African American	1.91[1.05, 3.33]	0.027*
Hispanic/Latino	1.64 [0.99, 2.68]	0.049*
Multiracial/other	1.23 [0.63, 2.23]	0.525
Below poverty threshold	0.83 [0.50, 1.34]	0.469
No high school diploma	1.60 [0.95, 2.59]	0.064

*Significant at *p* < .05

*Abbreviations*: AOR, adjusted odds ratio; CI, confidence interval

Although causation cannot be established in our analysis, there is a strong relationship between young people having a history of arrest and their mental health concerns, especially substance abuse and the risk of suicide.

## Discussion

Young people with a history of arrest display disparities among their mental health concerns. According to this study, it is more visible among women who encountered the criminal justice system through arrest, which was associated with increased odds ratios for all analyzed mental health concerns. Among men, an arrest history was associated with an increased probability of substance use, suicidal ideation, and suicide attempt. Results for women corroborate findings from a 2017 study on mental and physical disparities among young women with an arrest history that also found an association between arrest history and increased odds of having mental health disorder (Fedock & Sarantakos, 2017). Although causation cannot be established, there is a strong association between mental health outcomes and arrest history.

Of most concern, a history of arrest was strongly related to significantly higher rates of substance use among both gender groups and was more strongly pronounced among women. Previous research found that substance use disorder (SUD) substantially increased the risk of involvement in the criminal justice system; moreover, concurrent mental illness and SUD elevated the odds of arrest (Magee et al., 2021; Nkemjika et al., 2022; Prince & Wald, 2018). This study adds to the literature by providing findings for the young population, separately for men and women.

The next concerning outcome was suicide risk (suicide attempt and suicidal ideation), of which odds greatly increased for people with a history of arrest and, similarly to SUD findings, were higher for women. This study builds upon previous research that also found an association between arrests and increased risk of suicide (Bryson et al., 2021a, b; Cook, 2013). Additionally, a history of arrest was associated with a higher risk of SMI and depression among women. Findings for these two variables were not statistically significant among men. These results also build upon previous research finding that women in the criminal justice system experience mental health problems more often than men (Maruschak et al., 2021).

These findings have implications for public health and health policy. Mental health and criminal justice encounters are interrelated. On the one hand, individuals with mental illnesses are more likely to get arrested. On the other hand, incarceration adversely affects already poor mental health. A study on young adults found that approximately one-third of individuals had mental health or SUD diagnoses two years before arrest (Magee et al., 2021).

Moreover, people with multiple mental health conditions or SUD diagnoses had higher odds of repeated arrest (Magee et al., 2021). Lack of access to appropriate mental health treatment or nonadherence to medications for SMI can also increase one’s odds of repeated encounters with the criminal justice system (Bhatta et al., 2021). Therefore, providing arrestees with mental health diagnoses and appropriate treatment for those suffering from mental illness is essential to prevent recidivism and improve their resocialization process. Mental health concerns should be approached from multiple perspectives. Interventions implemented among individuals residing in correctional facilities, before their release from an institution and at the point of arrest, might effectively address their mental health.

Substance use care in the criminal justice system is insufficient and fragmented (Davis et al., 2018). Research shows that providing the incarcerated population suffering from SUD with an appropriate treatment might decrease their risk of substance use and overdose and lower mortality (Malta et al., 2019). Moreover, treated individuals have a lower likelihood of recidivism and higher odds of adherence to treatment and gaining employment after incarceration (Malta et al., 2019). Another approach could be pre-release enrollment in a health insurance plan. One study (Burns et al., 2022) found that assistance with pre-release enrollment in Medicaid increased healthcare utilization, especially substance use care, among the released population (Burns et al., 2022).

Another study (Alarid & Rubin, 2018) examined recidivism outcomes among adults with mental illness arrested for misdemeanor offenses who voluntarily agreed to stabilize on medication and report to a community-based outpatient mental health clinic. Authors have found that participants had fewer rearrests and spent fewer days in jail following discharge from the diversion program than the year before participation (Alarid & Rubin, 2018). Therefore, expanding diversion programs might effectively provide the needed care in the criminal justice system.

## Limitations

Measures of arrest history, education level, income level, and mental health outcome variables were self-reported and may be subject to social desirability, introspective ability, or recall bias. Moreover, although the National Survey on Drug Use and Health is nationally representative, it excludes individuals experiencing homelessness who do not use shelters, active military personnel, and residents of institutional group quarters such as jails, nursing homes, mental institutions, and long-term care facilities. Since some of the excluded population groups may have higher arrest history and mental illness rates, the actual response rate for these variables might be higher.

## Conclusions

This study examined relationships between a history of arrest and five mental health outcomes. We found a strong relationship between young people having a history of arrest and experiencing mental health concerns. Moreover, among young women, an arrest history affected all mental health concerns, while it was associated with only SUD nand suicide among young men. Providing arrestees with appropriate mental health care at the point of arrest, in the correctional facility, and before a release from incarceration would benefit them and the criminal justice system by decreasing the odds of repeated arrests.

## Data Availability

Data is not available due to privacy and ethical restrictions.
